# Soft Sensing of LPG Processes Using Deep Learning

**DOI:** 10.3390/s23187858

**Published:** 2023-09-13

**Authors:** Nikolaos Sifakis, Nikolaos Sarantinoudis, George Tsinarakis, Christos Politis, George Arampatzis

**Affiliations:** Industrial and Digital Innovations Research Group (INDIGO), School of Production Engineering and Management, Akrotiri Campus, Technical University of Crete, 73100 Chania, Greece; nsifakis@tuc.gr (N.S.); nsarantinoudis@tuc.gr (N.S.); gtsinarakis@tuc.gr (G.T.); cpolitis1@tuc.gr (C.P.)

**Keywords:** industrial monitoring, early fault detection, soft sensors, deep learning, industrial processes, oil refinery

## Abstract

This study investigates the integration of soft sensors and deep learning in the oil-refinery industry to improve monitoring efficiency and predictive accuracy in complex industrial processes, particularly de-ethanization and debutanization. Soft sensor models were developed to estimate critical variables such as the C2 and C5 contents in liquefied petroleum gas (LPG) after distillation and the energy consumption of distillation columns. The refinery’s LPG purification process relies on periodic sampling and laboratory analysis to maintain product specifications. The models were tested using data from actual refinery operations, addressing challenges such as scalability and handling dirty data. Two deep learning models, an artificial neural network (ANN) soft sensor model and an ensemble random forest regressor (RFR) model, were developed. This study emphasizes model interpretability and the potential for real-time updating or online learning. The study also proposes a comprehensive, iterative solution for predicting and optimizing component concentrations within a dual-column distillation system, highlighting its high applicability and potential for replication in similar industrial scenarios.

## 1. Introduction

The combination of soft sensors and deep learning is starting to change industrial practices in a major way, especially in oil refineries, by improving monitoring efficiency and prediction accuracy. Soft sensors are advanced models that use control theory and data-handling techniques to compute complex values from various types of measurements measurements [1]. These systems work by merging statistical methods, including principal component analysis (PCA) and the partial least squares (PLS) method, with modern AI tools such as deep neural networks (DNNs) [2,3,4,5].

The development and application of soft sensors have gained significant attention in various industries, including bioprocessing [6,7]. Soft sensors play a crucial role in estimating process variables, such as temperature, pressure, and flow rate, based on available data and mathematical models [8,9,10,11]. They contribute to the digitalization of manufacturing processes, leading to increased transparency, lower risks, and improved efficiency [12,13]. The merging of these technologies allows for the real-time monitoring of hard-to-reach key performance indicators (KPIs), significantly cutting costs by reducing the need for expensive, traditional hardware [14,15,16].

Deep learning, a specialized branch of AI known for its capability of managing large datasets and recognizing complex patterns, further improves soft sensor capabilities. The use of deep learning techniques, such as convolutional neural networks (CNNs), recurrent neural networks (RNNs), and long short-term memory (LSTM) networks, has proven effective in predicting nonlinear and time-dependent industrial processes [17,18,19,20]. These techniques are especially useful in oil-refinery processes, such as for identifying the composition of C4 hydrocarbons in distillation columns [21,22,23]. Such uses show how these systems can predict complex process variables accurately, enhancing real-time analysis and enabling data-driven decision-making [24,25,26,27].

However, several technical challenges pose serious obstacles [28]. Making sure that these models are reliable under changing operating conditions, dealing with data security issues, and avoiding the risks of model overfitting are areas needing urgent attention [29,30]. In addition, the successful use of these technologies needs careful planning regarding data handling, feature selection, and effective model validation techniques [31,32,33]. Dealing with these challenges demands a deeper understanding of both soft sensor design and the underlying deep learning mechanisms. This study aims to dive deeper into these issues, offering easy-to-understand insights into the impact and potential of soft sensors and deep learning on industrial oil-refinery processes.

In the examined refinery, particularly in the liquefied petroleum gas (LPG) purification process, maintaining the final product within specifications is paramount. Historically, the LPG that accumulated in the tank was subject to periodic sampling and laboratory analysis at sparse intervals, typically once daily. Subsequently, these findings were communicated to the process engineers, who would make the necessary adjustments to the control parameters of each process unit. The considerable time lapse between sampling and obtaining results implied that any off-specification deviations would necessitate a roughly 24-h window for detection, subsequently requiring corrective measures. These may include elevating the LPG purification standards for blending with off-specification tanks, re-purifying the entire tank, or, in extreme cases, discarding the product entirely. The soft sensors under investigation are designed to provide process engineers with predictive insights into the anticipated concentrations of C2 and C5, thereby enabling preemptive corrective actions before the manifestation of any issues. It is important to note that these soft sensors are intended to complement, rather than replace, traditional laboratory analyses, serving as an auxiliary tool for process engineers in conjunction with conventional laboratory testing.

In the context of this research work, a soft sensor model was developed that simultaneously models the crucial chemical processes of de-ethanization and debutanization in the oil refinery industry. The data used to train and evaluate the deep learning soft sensor model came from various online sensors that are currently installed in a refinery. The purpose of the developed soft sensor is to predict the C2, C5, and Q (energy demand) contents of liquefied petroleum gas (LPG) after it has passed through both distillation columns, and to predict the energy consumption of the columns. These predictions are derived using certain process variables, namely, pressure and temperature, as well as the mixture flow from the reboiler, which can be controlled by the refinery staff. It is, therefore, possible to use the model to simulate how changes in the manipulated variables of the distillation columns can affect the final product. By leveraging advanced deep learning techniques, this versatile and dynamic approach for the prediction and optimization of component concentrations within a dual-column distillation system is proposed.

The remainder of the paper is organized as follows. Section 2 discusses the state of the art in relation to the specific issue of implementing soft sensors in the oil-refinery industrial sector. The case study description, methodology, and materials utilized are all shown in Section 3. The findings of this research are given in Section 4 and are discussed in Section 5. The study’s conclusions and recommendations for the future are presented in Section 6.

## 2. State of the Art

Soft sensors, also known as virtual sensors, have been pivotal in the oil refinery industry, presenting a cost-effective, reliable solution for predicting quality variables that are otherwise difficult or expensive to measure directly [34]. These innovative tools have transitioned from basic mathematical models to complex systems that leverage advanced data-driven techniques such as AI and ML. AI and ML technologies, notably Gaussian processes, fuzzy logic systems, and neural networks, have played a significant role in improving prediction accuracy, handling missing data effectively, and augmenting the adaptability of soft sensors to shifting data sources [34,35,36,37,38].

Recent research has focused extensively on the development and application of soft sensors within oil refineries. For example, a soft sensor model based on Kaizen programming was used to estimate C4 hydrocarbon composition in a distillate stream in an oil refinery, showcasing superior performance compared to a Kriging-based model. Another significant innovation was the introduction of the Spectral AutoML approach for the development of process analytical technology (PAT) soft sensors, which demonstrated excellent performance in predicting diesel fuel properties [39].

Designing and implementing soft sensors involves various processes, such as data preprocessing, algorithm-based model construction, ensemble modeling, and model validation [40,41,42]. Researchers have proposed hybrid models that combine AI techniques such as fuzzy logic systems and neural networks to handle complexity and enhance predictive power [43,44]. Additionally, one largely unexplored domain within the soft sensor field is the incorporation of automated model selection and hyperparameter tuning techniques, such as AutoML. Innovations such as Spectral AutoML, which incorporates pre-processing, band selection, resolution definition, hyper-parameter tuning, and model estimation, are helping to optimize inferential models [45] These techniques could streamline the development of soft sensor models, optimizing their performance and broadening their accessibility to industry professionals who lack an extensive background in ML.

Dealing with “dirty data”, characterized by missing values, inconsistent formatting, or noisy measurements, also remains a complex issue. Current ML models often assume the availability of clean and well-structured data, which is not always the case in real-world industrial settings [46,47]. However, several challenges persist, such as the integration of soft sensors into processes governed by first-principle models giving rise to concerns related to data security and privacy, given the heavy reliance of soft sensors on data [48,49]. Furthermore, despite the successful application of various models in small-scale or laboratory conditions, the scalability of these applications to full industrial processes remains unexplored [50].

Therefore, understanding the interpretability of AI/ML-based soft sensor models is a critical aspect that needs to be addressed. The “black box” nature of these models, while delivering high accuracy, can make it difficult to interpret the results. Transparency in these systems is vital for building trust. The potential for real-time updating or “online learning” in the context of soft sensors is another under-researched area. Given the dynamic nature of industrial processes, a soft sensor model’s ability to learn and adapt in real time could dramatically enhance its performance. As technology continues to evolve, soft sensors are poised to revolutionize oil refinery operations, offering the potential for increased efficiency, significant cost reductions, and operational efficiency enhancement.

This research addresses key challenges in the use of soft sensors in the oil refinery industry, using the latest techniques and tools to enhance this important field of study. The issue of scalability is tackled by testing our soft sensor model using data from real-life refinery operations, showing its effectiveness in a real industrial setting. The problem of dealing with “dirty data”, which refers to data with inconsistencies and errors, is addressed by designing a soft sensor model to handle these issues. This feature makes the model more accurate and reliable in real-world scenarios. A comprehensive, iterative, and adaptable solution for predicting and optimizing component concentrations within a dual-column distillation system is proposed, highlighting its high applicability and replicability.

Understanding how the model makes its predictions, also known as model interpretability, is a critical aspect that this research emphasizes. By making the model’s decision-making process clearer, trust is built and the refinery workers’ and stakeholders’ ability to make informed decisions is facilitated. Given the dynamic nature of industrial processes, a soft sensor model’s ability to learn and adapt in real time could dramatically enhance its performance. With ongoing technological advancements, the transformative potential of soft sensors in oil refinery operations becomes increasingly evident, promising substantial improvements in efficiency and cost-effectiveness. This research represents a significant advancement in the field of soft sensor development. By addressing key challenges such as scalability, “dirty data,” and model interpretability, and by proposing a dynamic and adaptable solution for managing component concentrations, this article strongly contributes to the ongoing evolution of soft sensing in oil refinery operations.

## 3. Materials and Methods

### 3.1. Case Study Description

Refineries, industrial facilities processing crude oil into products such as naphtha, petrol, bitumen, and LPG, utilize distillation columns for fractional distillation, thereby separating oil derivatives based on their boiling points. These initial products typically undergo further processing for later sale and usage. LPG, a petroleum derivative, is primarily a hydrocarbon mixture. Its composition shifts with the season, containing more propane in winter and more butane in summer, although it generally consists of both. Small quantities of propylene, butylene, and other hydrocarbons are also present.

The data in this paper come from a refinery with an annual crude oil processing capacity of over 11 million tons, producing diverse products including LPG, various fuels, lubricating oils, paraffins, and different asphalt specifications. The refinery’s structure is divided into several units, each responsible for producing a specific product, including LPG, produced via six methods:

1. Atmospheric or crude distillation unit.
2. Hydrocracker unit.
3. Fluid catalytic cracking unit.
4. Delayed coker unit.
5. Maximum quality diesel unit.
6. Platformer unit.

In post-production, the LPG from each unit undergoes purification in degasifiers and LPG DEA units to remove hydrogen sulfide (H_2_S) and carbonyl sulfide (COS). This step is completed using diethanolamine (DEA), resulting in purified LPG that is ready for storage. Notably, the hydrocracking unit product also needs de-ethanization, while the platformer product is sent directly to the storage tanks.

**Figure 1:**
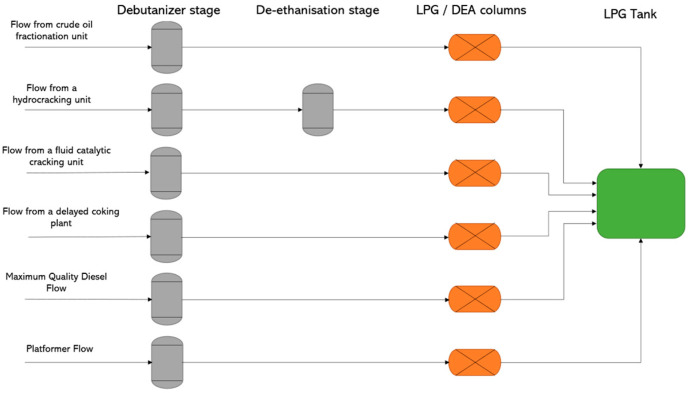
Simplified illustration of the LPG purification device.

In this study, a deep-learning soft sensor was developed to simulate a dehydrator/de-ethanizer system purifying LPG from the hydrocracking units (Figure 1). This system inputs a mixture of LPG and light straight run naphtha (LSRN) (C5) into a degasifier. Since C5 is a gasoline component, it is retained in the LSRN for further processing, while being removed from the LPG. Controlling this C5 content within the LPG is achieved by adjusting the dehydration column process parameters. The process leads to a by-product that is used internally in the refinery for energy, including running the heating furnaces. Subsequently, the de-ethanization columns remove C2 from the LPG mixture, with the process parameters adjusted to control the quality of the resultant product.

### 3.2. Data Acquisition & Pre-Processing

#### 3.2.1. Data Acquisition

The data in the study originated from an oil refinery equipped with a variety of real-time sensors. These sensors were strategically situated at critical points to measure specific process parameters. There were four main sensor categories: temperature, pressure, flow, and substance concentration. Within the degasser under analysis, sensors measured the temperature at various points, including the column trays, the top and bottom of the column, and the entry and return points of the LPG mixture. The flow rates at the column inlet, the reheater, and the diesel used in the reheater were recorded by flow sensors. The pressure was only measured at the column top. The debutanizer had a similar sensor arrangement. C5 content was measured at the degasser outlet, while C2 content was monitored at the de-ethanizer outlet.

Although these sensors generated a great deal of data, not all the parameters they measured could be considered and controlled. The engineers responsible for the production process could only adjust the peak temperature, the peak pressure, and the flow of the LPG mixture from the reheater. Therefore, the deep-learning soft sensor that had been developed to simulate the two-column distillation system only accepted these data as inputs. This approach facilitated the simulation of potential changes in LPG quality based on the modifications that could be made by the refinery staff. The soft sensor’s outputs were the C2 and C5 content in the LPG, substances considered impurities that needed to be within specific limits.

Additionally, the soft sensor predicted the system’s energy usage. Both the dehydration and de-ethanization columns employed heat exchangers to meet their energy needs. While there was no direct way to quantify the system’s energy consumption, the available data provided valuable insights. This included data on the diesel flows utilized in the heat transfer within the two columns’ heat exchangers, as well as the temperature of the diesel at the inlet and outlet of these heat exchangers.

Using the first law of thermodynamics, the system’s energy requirements can be approximated as the heat energy loss from the diesel used in the heat exchangers:

(1) $$\dot{Q}=\dot{m}C_{p}\Delta T$$

where $\dot{Q}$ is the heat transfer rate, m is the mass transfer rate of the substance, *C_p_* is the specific heat of the substance, and Δ*T* is the temperature difference of the substance between the inlet and the outlet of the heat exchanger. The substance in the present case is diesel. Although no data are available on the mass transfer rate of diesel in the system, by using the following equation:

(2) $$\rho =\frac{m}{V}=\frac{\dot{m}}{\dot{V}}\to \dot{m}=\rho \dot{V}$$

where *ρ* is the density and *V* is the volume transfer rate, Equation (1) becomes:

(3) $$\dot{Q}=\rho \dot{V}C_{p}\Delta T.$$

The values for *ρ* and *C_p_* are provided by the refinery and, in combination with the data for the inlet, outlet, and flow temperatures of the diesel in the heat exchanger, it is possible to calculate the energy consumption of the system.

#### 3.2.2. Data Pre-Processing

The dataset utilized in the model’s development spanned two years, with a sensor timestep for every minute. To ensure the effectiveness of the deep-learning soft sensor, the data needed validation and cleaning, ensuring that only useful information was retained. Initial data were provided by the refinery in the form of Excel files in “Unix time” format, yielding a total of nine files. Six of these files informed the model inputs, while the remaining three informed the outputs.

The initial stage of data pre-processing was conducted to identify and eliminate duplicate entries, as well as instances where the sensors neglected to record necessary data. Anomalies, whether one-off incidents or more extended periods of recording failure, possibly due to sensor malfunction, were meticulously examined. Every discrepancy was subjected to a rigorous evaluation process. The data management strategy employed a two-pronged approach: either omitting the erroneous time frames across all datasets or using the mathematical method of linear interpolation to insert plausible data points where none previously existed.

Further refinement was undertaken by locating and removing instances of illogical data, particularly those where the sensors had recorded negative values for inherently positive quantities, such as the concentration of C2 in the LPG mixture. This thorough and diligent pre-processing stage underlines the importance of data integrity and ensures that the dataset provides a solid foundation for the subsequent steps in the model development process.

To identify and remove outliers, the interquartile range (IQR) method was employed. Sensor data were organized in ascending order and divided into quartiles, identifying the Q1 and Q3 points. Q1 is the point where 25% of the data is lower and Q3 is the point where 75% of the data is lower. The gap between quartiles, denoted as H = Q3 − Q1, was calculated. Data values of less than Q1 − 1.5H and greater than Q3 + 1.5H were deemed outliers and were thus removed. Lastly, the remaining data were collected and normalized, using the following equation:

(4) $$x_{norm}=\frac{x-\mu }{\sigma^{2}}$$

where *μ* is the mean and σ^2^ is the standard deviation, thus completing the pre-processing of the data. Table 1 shows the range of values recorded by the sensors after the preprocessing stage.

**Figure 2:**
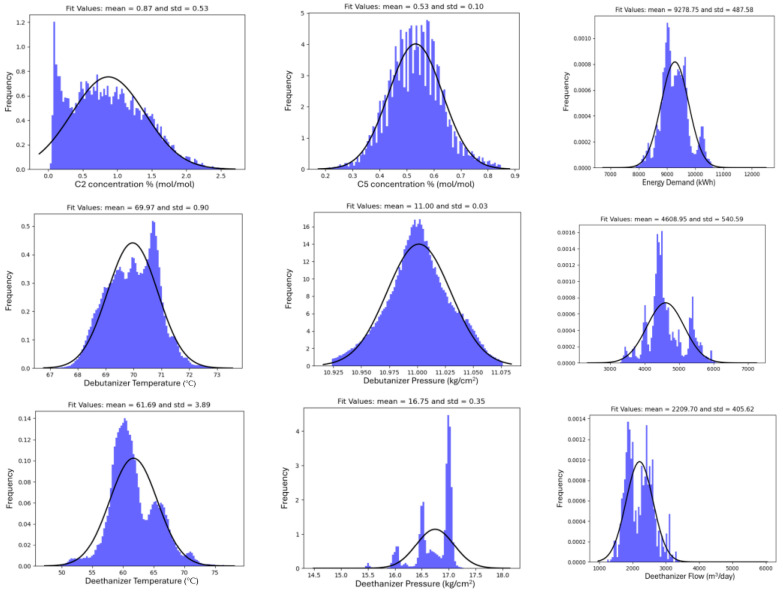
Data distribution across the different variables.

The new “cleaned” dataset contained over half a million time-stamped measurements for a range of variables that are, presumably, strongly related to the industrial process. These include the variables for C2 and C5 concentrations, with mean values around 0.87 and 0.53, respectively. Temperature readings (Temp_But and Temp_Et) exhibited means of around 69.97 and 61.69, indicating varied temperatures over time. The pressure measurements (Pres_But and Pres_Et) appeared relatively stable, with mean values of 11.00 and 16.75, respectively. Other parameters, Reb_But and Reb_Et, showed significant variability, with mean values of around 4608.95 and 2209.70. These preliminary insights suggest a complex dataset, capturing a dynamic process with significant variations over time. The data distribution for each parameter is shown in Figure 2, which supports the findings mentioned above.

### 3.3. Soft Sensor Modeling

The objective of this study is to establish an optimal deep-learning soft sensor model for a two-column distillation system using both ANN and Random Forest Regression (RFR) methods. Optimal performance is characterized by the lowest error found on previously unseen data. The optimal model will have the best configuration of hidden layers and neurons. The methodology used to optimize the network relies on a “train–validation–test split” and an iterative process for determining the best architecture.

In simpler terms, the “train–validation–test split” approach works by dividing the starting dataset into three smaller sets. The first set, the training set, helps the model to understand and learn the parameters. The validation set then steps in to check these learned parameters and fine-tune them to reach the best possible performance. Finally, the test set is used after the model has finished learning, to see how well the model can make predictions using new data that it has not encountered previously.

Finding the ideal architecture of the ANN deep learning soft sensor model began with a structure that includes a single hidden layer with just two neurons. After teaching the model using the training and testing data, the Root Mean Squared Error (RMSE) was calculated using the validation data. This process was repeated, each time doubling the neurons in the hidden layer until it reached 256 neurons. At this point, an extra hidden layer was added with two neurons, and the process continued. When determining the model’s hyperparameters, a learning rate of 0.001 and a batch size of 512 for the final model were chosen through a process of trial and error. These settings can have a considerable impact on the overall time that it takes to train the model. Generally, using larger batch sizes can speed up training times, but this may lead to larger prediction errors. To save time and effort when testing new architectures, a large batch size was chosen to start with, then if the results looked promising, we tried other batch sizes to achieve the lowest possible error.

To control the number of times that the model goes through the training data (epochs), early stopping and checkpoint methods were used. Early stopping is a way to stop the training process if a set number of epochs have gone by without any improvement in the model’s errors. Checkpoints, on the other hand, help to remember those parameters that have provided the best modeling results before stopping the training. These methods work together to prevent overfitting and to save the optimal model parameters.

Therefore, two random forest models were deployed. These models, which function by constructing multiple decision trees during training and then yielding the mean prediction, were trained and then used to make predictions on the validation set. Model performance was evaluated using R^2^ scores and RMSE, with the differences between the predicted and actual values being visually displayed through residual plots and histograms.

Lastly, the contribution of each predictor to the models was analyzed and visualized. This process revealed those variables significantly influencing the model predictions, gauged through the improvement that each attribute split point provided, weighted by the number of observations for which the node was responsible.

### 3.4. Assumptions

The study and the modeling techniques used above have been based on several assumptions, including:

- The random forest model typically assumes that the predictors (features) are independent of each other;
- The iterative process of increasing neuron count and hidden layers would lead to an optimal or near-optimal architecture;
- The selection of hyperparameters, such as the learning rate and batch size, is often a balance between computational efficiency and model performance. It is assumed that the chosen hyperparameters are the most appropriate for this task;
- The use of early stopping and checkpoints is based on the assumption that overfitting occurs when no improvement is observed over a specific number of epochs.

## 4. Results

For the purposes of this study, two different types of deep learning models were established, studied, and evaluated:

(a) An ANN soft sensor model

ANN models come with the ability to learn and adapt to even the most complex and non-linear relationships. This attribute makes them a go-to solution when tackling various real-world scenarios that may involve intricate and non-linear patterns. In the context of an oil refinery, there is a wealth of data available. ANNs, known for their ability to handle large datasets smoothly, are well-suited. Furthermore, once an ANN model is well-trained, it is quite nimble in providing outputs, enabling real-time predictions. Such quick predictions are vital in a functioning refinery, as they aid in promptly monitoring parameters such as impurity concentrations, thereby ensuring operational efficiency.

(b) An Ensemble RFR model

The ensemble random forest model was another critical component of the predictive framework used. Known for its robustness and accuracy, random forest, an ensemble learning method, often delivers highly accurate predictions while guarding against overfitting. Such models work by constructing multiple decision trees during training and providing an averaged output, which, in turn, reduces bias, decreases variance, and generally results in superior performance. Similar to ANNs, random forests are excellent at dealing with many input features without succumbing to overfitting, making them an ideal choice for high-dimensional data. A highlight of using random forests is their ability to offer a peek into which features hold more importance in predictions. This valuable insight can help decipher the underlying process and refine future models. In a nutshell, the strong suit of random forest models, which includes robustness, accuracy, and interpretability of features, perfectly complements the flexibility and real-time prediction capabilities of ANN models.

### 4.1. ANN Soft Sensor Results

**Figure 3:**
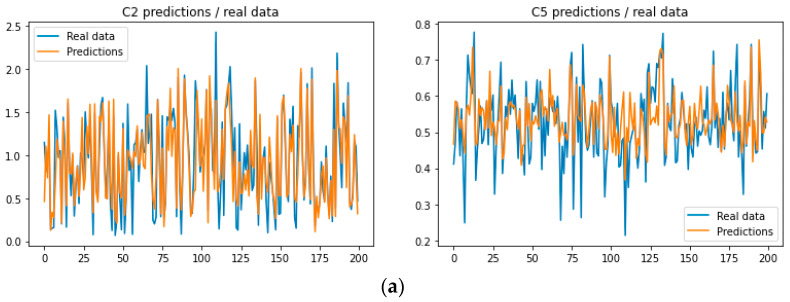

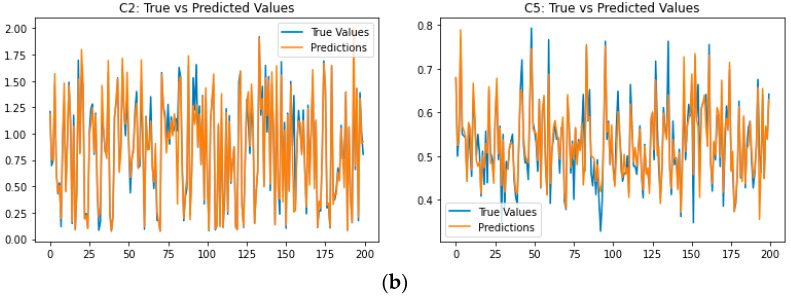
(**a**) Predictions versus actual values for the ANN soft sensor model for both C2 and C5, (**b**) predictions versus actual values for the RFR soft sensor model for both C2 and C5.

**Figure 4:**
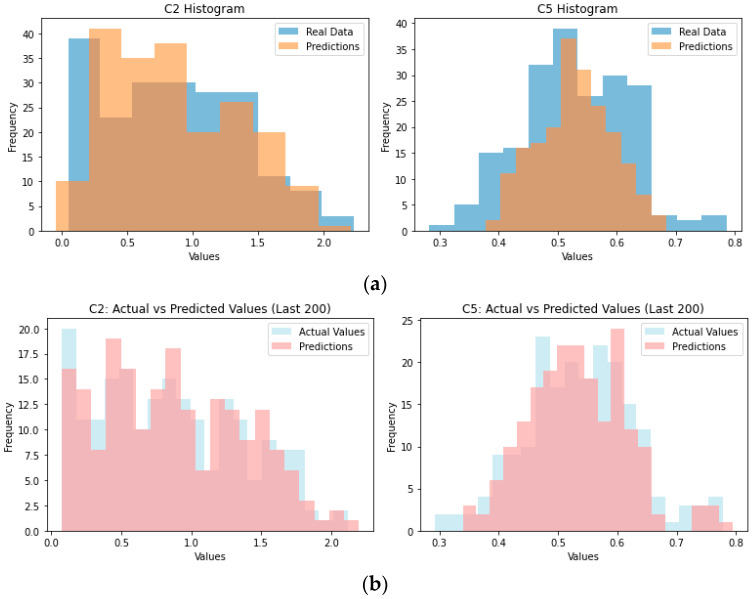
Histograms of C2 and C5 values (actual versus predictions) for the (**a**) ANN model and (**b**) the RFR model.

The ANN soft sensor models for the C2, C5, and energy demand outputs have produced interesting and relatively accurate predictions. Specifically, in this study, an ANN soft sensor was employed for the prediction of three variables—C2, C5, and Q—within a complex system. The ANN was trained on a comprehensive dataset, with its performance evaluated using RMSE, MAE, and R^2^ metrics. The ANN demonstrated high predictive accuracy for C2 (Figure 3a) and Q (Figure 4a), as indicated by low RMSE and MAE, along with high R^2^ scores. Conversely, for C5 (Figure 3a), the model exhibited a lower level of precision, marked by a lower R^2^ score. This analysis highlights the utility of the ANN model in predicting C2 and Q and underscores the need for further model optimization for “C5” prediction.

As can be seen from Figure 3a, the ANN soft sensor model exhibits significantly good performance for the prediction of C2, while the predictions for C5 are not that accurate. The R^2^ index values are 0.7976 and 0.3618, respectively.

Table 2 presents the most important metrics for C2 and C5 according to the models’ outcomes. These values are lower than the ones from the ANN model, which indicates that the RFR model leads to better predictions.

**Figure 5:**
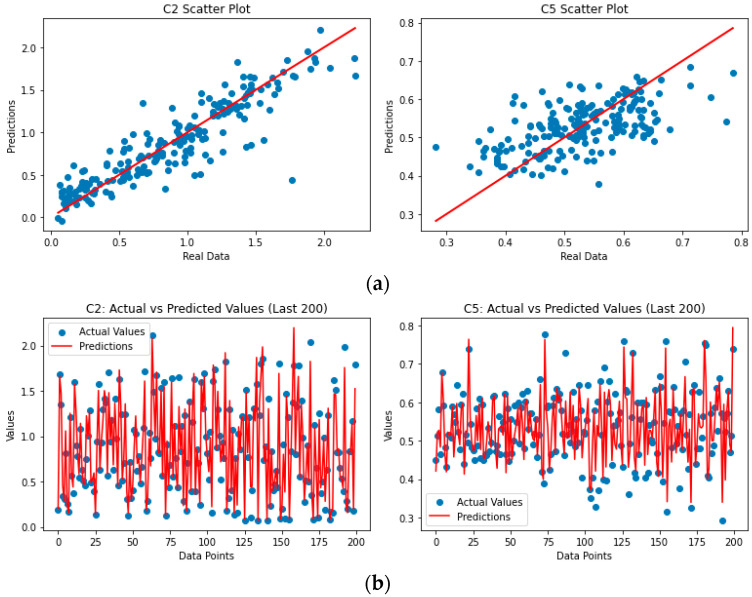
Scatter plots of C2 and C5 values (actual versus predictions) for (**a**) the ANN model and (**b**) the RFR model.

Lastly, the histograms for the actual values for C2 and C5 and the model’s predictions can be used to support the previous statements by observing the distribution of values (Figure 4a). Similarly, the scatter plots in Figure 5a indicate the same outcomes, showing the better performance of the C2 model compared to the C5 model.

**Figure 6:**
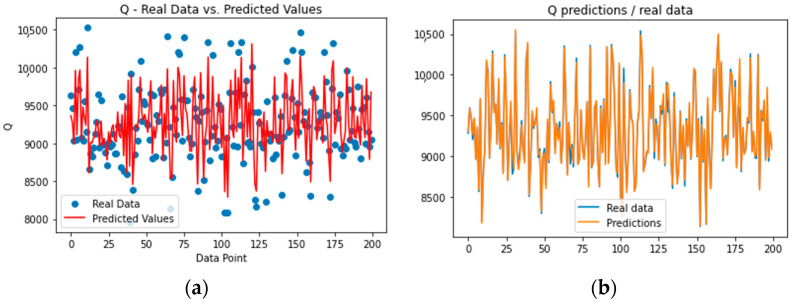
Predictions versus actual values of the Q values for (**a**) the ANN soft sensor model and (**b**) the RFR soft sensor model.

Correspondingly, for the Q predictions, Figure 6a shows the actual measurements in comparison with the predictions from the established model, which seem to be satisfactory. Specifically, the evaluation metrics are presented in Table 2; the predicted values are near to the actual ones and the errors can be considered low compared to the average value of Q and the standard deviation.

**Figure 7:**
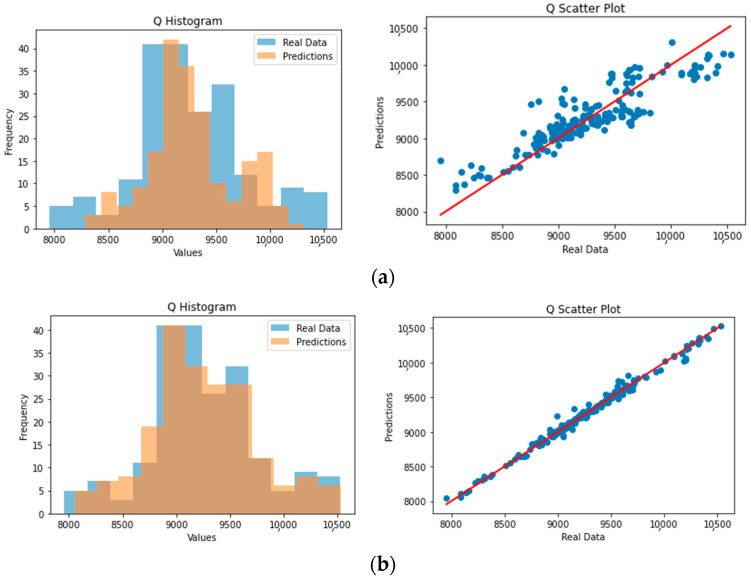
Histogram and scatter plot of Q values (actual versus predictions) for (**a**) the ANN model and (**b**) the RFR model.

As can clearly be observed in Figure 6a and Figure 7a, the upper and lower bounds of the energy demand were not accurately predicted by the established model, a solution for which was attempted using the RFR soft sensor model, as described in the next subsection.

### 4.2. RFR Soft Sensor Results

The RFR soft sensor models for the C2, C5, and energy demand outputs have produced attractive and adequate predictions. Specifically, an RFR soft sensor was employed for the prediction of three variables—C2, C5, and Q—for the oil-refinery system. The ANN was trained on a comprehensive dataset, with its performance evaluated using RMSE, MAE, and R^2^ metrics. The RFR demonstrated very high predictive accuracy for C2 (Figure 3b) and Q (Figure 4b), as indicated by the low RMSE and MAE values, along with high R^2^ scores. Conversely, for C5 (Figure 4b), the model exhibited a relatively high level of precision, marked by a lower R^2^ score. This analysis highlights the utility of the RFR model in accurately predicting C2, C5, and Q.

Lastly, the histograms for the actual C2 and C5 values and the model’s predictions can justify the above by observing the distribution of values (Figure 4b). Similarly, the scatter plots in Figure 5a indicate the same outcomes, showing the better performance of the C2 model compared to the C5 model.

Similarly, for the Q predictions, Figure 6b shows the actual measurements in comparison with the predictions from the established model, which seem to be highly accurate. Specifically, the evaluation metrics are presented in Table 2; the predicted values are almost equal to the actual values and the errors can be considered scarce.

As can clearly be observed from Figure 7b, the upper and lower bounds of the energy demand were accurately predicted by the established model in this case (as opposed to the previous ANN model), which indicates a highly accurate model.

The feature importance values for the C2 model indicate that the most important features for predicting C2 concentration are “C2” and “Temp_Et”, with importance values of 0.39 and 0.36, respectively. These two features contribute significantly to the model’s prediction, accounting for approximately 75% of the prediction. The other features, “Pres_But”, “Temp_But”, “Reb_But”, and “C5”, have lower importance values ranging from 0.02 to 0.09, indicating that they have a lesser impact on the C2 prediction. For the C5 model, the most important features for predicting C5 concentration are “Temp_Et” and “Temp_But”, with importance values of 0.28 and 0.24, respectively. These two features contribute most substantially to the model’s prediction, accounting for more than 50% of the prediction. The other features, “Pres_But”, “Reb_But”, “C2”, and “C5”, have lower importance values, ranging from 0.05 to 0.17, indicating that they have a lesser impact on the C5 prediction.

As for the energy consumption, the highest feature importance is for Reb_But (0.767), indicating that the energy consumption is highly influenced by the reboiler. This is followed by Reb_Et (0.097) and Temp_Et (0.052), which have the next-highest importance values. The other features, Temp_But, Pres_But, and Pres_Et, have relatively lower importance values, indicating that they have less influence on energy consumption. Understanding these feature importance values can help in optimizing the process by focusing on the most influential parameters, which, in this case, is the reboiler.

## 5. Discussion

The key reason behind the appropriateness of the two models lies in their intrinsic ability to handle complex and non-linear systems, making them suitable for applications in industries such as oil refining, which involve a multitude of interacting variables. The estimation of these variables is typically a complex task that involves multiple parameters and interactions. The two models provide high accuracy, even in cases where the underlying process is not clearly understood or the relationships between the variables are non-linear. Lastly, the two models provide insights into feature importance, this being exceptionally helpful in refining the sensor models or understanding which variables have a significant impact on the output.

### 5.1. ANN Soft Sensor

The model’s performance appears to vary across the different variables. Its ability to predict C2 and Q is satisfactory, while it seems to struggle somewhat with “C5”. The extent to which this performance is deemed acceptable would depend on the specific context and objectives of the modeling exercise, including the acceptable level of error and how this model’s performance compares to that of other models or benchmarks.

For C2, the model exhibits a substantial degree of predictive power. The discrepancy between the model’s predictions and the actual values, as measured by the RMSE, is less than half the standard deviation of the observed C2 values. The average error, represented by the MAE, further corroborates the model’s efficacy in predicting C2. Moreover, the model’s ability to explain the variance in C2 is quite high, with an R^2^ score of 0.7976, which indicates that approximately 79.76% of the variance in C2 can be accounted for by the independent variables included in the model.

The model also performs reasonably well for Q. With an RMSE of 249.17 kW and an MAE of 193.77 kW, the model’s predictions for Q are generally close to the actual values. Furthermore, the model explains a substantial portion of the variance in Q, as reflected in the R^2^ score of 0.7396.

However, the model’s performance dips when predicting C5. While the RMSE and MAE for C5 are less than the standard deviation of the observed C5 values, indicating relatively accurate predictions, the R^2^ score is significantly lower at 0.3618. This suggests that the model’s ability to explain the variance in C5 is not as strong, pointing to potential areas for improvement in the model.

### 5.2. RFR Soft Sensor

The RFR model showcases a beyond-satisfactory predictive ability for C2 and Q, while its performance for C5, though reasonable, leaves room for improvement.

In the case of C2, the RFR model exhibits a considerable level of predictive precision. The RMSE, a measure of the average discrepancy between the model’s predictions and the actual values, is significantly less than half the standard deviation of the observed C2 values. This, in conjunction with the MAE, which represents the average error, corroborates the model’s robustness in predicting C2. Further supporting this finding is the high R^2^ score of 0.9561, suggesting that approximately 95.61% of the variance in C2 can be accounted for by the independent variables included in the model.

The model also exhibits an admirable performance in predicting Q, as indicated by an RMSE of 53.799 kW and an MAE of 33.562 kW, suggesting that the model’s predictions for Q align closely with the actual values. The model’s ability to explain a substantial portion of the variance in Q is reflected in the R^2^ score of 0.9878, a testament to the model’s strong predictive capability for this variable.

On the other hand, the model’s proficiency is slightly reduced when predicting C5. Although the RMSE and MAE for C5 are less than the standard deviation of the actual C5 values, indicating a degree of accuracy in the predictions, the R^2^ score is relatively lower at 0.8346. This implies that the model’s ability to account for the variance in C5 is not as strong, indicating potential areas for enhancement in the model.

### 5.3. Comparison of the Two Soft Sensor Models

Both models demonstrate reasonable predictive capabilities, with the RFR model outperforming the ANN model in predicting all three variables of C2, C5, and Q. The RFR model provides more accurate and reliable predictions, making it the more effective choice based on the given metrics.

In the case of C2, the RFR model outperforms the ANN model, providing more accurate predictions, with a lower RMSE and MAE, and a higher R^2^ score. The RFR model explains about 95.61% of the variance in C2, compared to the 79.76% explained by the ANN model. This suggests that the RFR model is more adept at predicting C2.

When predicting Q, both models perform well, but the RFR model edges ahead with an RMSE of 53.799 kW and an R^2^ score of 0.9878, indicating a very high level of accuracy. The ANN model, while satisfactory, falls short with a higher RMSE and a lower R^2^ score.

The prediction of C5 is where both models struggle. However, the RFR model again shows a stronger performance, with a lower RMSE and MAE and a higher R^2^ score. The ANN model’s R^2^ score for C5 is notably lower, suggesting less reliability in its predictions for C5.

**Figure 8:**
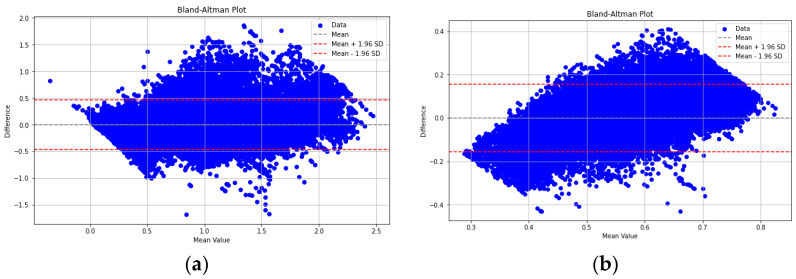
Bland–Altman plots for the (**a**) C2 and the (**b**) C5 models.

The Bland–Altman plots compare the true values of C2 (Figure 8a) and C5 (Figure 8b) with the predicted values of C2 and C5, respectively, from the established models. The C2 model has most of its points lying within the limits of agreement and has a smaller bias and narrower limits of agreement compared to the C5 model. This suggests that the C2 model is in better agreement with the actual values and is a better model. Additionally, the C2 model has a higher R^2^ score, lower RMSE, and lower MAE compared to the C5 model, further justifying its superiority against the C5 model.

In addition, according to the domain knowledge provided by the industry’s process engineering team, C2 removal has a greater effect on the final product and the crude oil that is refined has a higher concentration of C2 than C5. Thus, the C2 measurements consequently create a better dataset than C5 to produce the aforementioned models, and, thus, the performance is better regarding C2 predictions. The C2/C5 removal is also affected by the quality of the crude input; however, no information was recorded by the refinery. In cases where a crude input had a higher C5 concentration, exploiting these data will most likely lead to an improved model.

The ANN soft sensor model and the ensemble RFR model are essential tools for real-time analysis and data-driven decision-making in oil refineries. The ANN model is well-suited for complex scenarios and can provide real-time predictions for monitoring parameters such as impurity concentrations, ensuring operational efficiency. The ensemble random forest model is known for its robustness, accuracy, and ability to handle multiple input features without overfitting. These attributes make the random forest and ANN models highly complementary and suitable for integration into existing oil refinery systems.

The soft sensor models developed in this research provide predictive insights into anticipated concentrations of C2 and C5, enabling preemptive corrective actions before any issues arise. These soft sensors complement traditional laboratory analyses and serve as auxiliary tools for process engineers. The model predicts the C2, C5, and Q (energy demand) contents of LPG after passing through the distillation columns and also the energy consumption of the columns. The model’s real-time adaptability enhances its performance, fostering trust among refinery workers and stakeholders and facilitating informed decision-making.

This study emphasizes the importance of thorough data validation and cleaning in model development. A detailed pre-processing strategy ensures the robustness and integrity of the developed models. Moreover, the comprehensive evaluation using customized metrics underscores the models’ validity and applicability compared to prior research [51,52,53]. This study fills a significant gap in the literature regarding the deployment of soft sensors in oil refineries, as most previous studies were focused on other industrial applications. It not only addresses this deficit by formulating robust models for oil refineries but also advances the field by demonstrating enhanced performance compared to that in prior research. The adopted methodology, involving rigorous data pre-processing and exhaustive model performance evaluation, establishes a novel benchmark for soft sensor development in oil refineries and similar applications. Additionally, the results indicate that the developed models are either superior or equivalent to those in past studies, highlighting the efficacy of the methodology and setting a new standard for soft sensor development and their application in oil refineries and similar contexts [54].

## 6. Conclusions

This research investigates the potential of integrating soft sensors, or virtual sensors, with deep learning within the oil refinery industry. The primary objective is to enhance the efficiency of monitoring and the predictive accuracy of complex industrial processes, specifically de-ethanization and debutanization. Various soft sensor models have been developed, designed to estimate critical variables such as the C2 and C5 contents in LPG after distillation and the energy consumption of the distillation columns. These estimations are derived from controllable process variables, including pressure, temperature, and the mixture flow of the reboiler.

The study confronts real-world challenges by testing the soft sensor models using data from actual refinery operations, thereby addressing the issue of scalability. Moreover, the model has been designed to work effectively with inconsistent and erroneous data, known as “dirty data”, thereby improving its accuracy and reliability in practical scenarios. A significant aspect of this research is its emphasis on model interpretability. The aim is to elucidate the decision-making process of the model, thereby fostering trust among refinery workers and facilitating informed decision-making.

The research has led to the development of two deep learning models—an ANN soft sensor model and an Ensemble RFR model. The ANN model, with its ability to learn and adapt to complex and non-linear relationships, is adept at handling large datasets and providing real-time predictions. Conversely, the ensemble RFR model is renowned for its robustness and accuracy, often delivering highly accurate predictions while safeguarding against overfitting. The ANN soft sensor models have yielded promising predictions for the C2, C5, and energy demand outputs.

This research underscores the importance of understanding the interpretability of AI/ML-based soft sensor models. The potential for real-time updating or “online learning” in the context of soft sensors is identified as an area ripe for exploration. Model interpretability is pivotal in applications where understanding the basis of predictions is essential for decision-making and communicating the model’s functionality to non-technical stakeholders. While RFR provides some interpretability through its feature importance scores, it does not offer insights into the relationships between features and the target variable or the interactions between features, making it less interpretable than simpler models such as linear regression. Conversely, ANNs are often considered “black box” models due to their intricate structure and limited interpretability, which poses challenges in understanding specific predictions and communicating the model’s functionality. Oil refineries, characterized by their complexity and variability in terms of crude oil type, refinery configuration, operating conditions, and specific processes, often require models that can capture complex non-linear relationships and the interactions between variables. Traditional modeling approaches, such as physical models or linear regression models, may not adequately capture these complexities. In such scenarios, “black box” models such as ANNs and RFR can be particularly useful as they can capture these complex relationships without the need for explicit modeling of the underlying physical processes. Although these models have limitations in terms of interpretability, their ability to provide better predictive performance in complex systems with many variables and non-linear relationships can be particularly desirable in oil refineries, where accurate and reliable predictions are crucial for optimizing operations and ensuring product quality. Ultimately, the choice of model depends on the specific requirements of the application and the needs of the stakeholders, and there may be trade-offs between predictive performance and interpretability.

Given the dynamic nature of industrial processes, a soft sensor model’s ability to learn and adapt in real time could significantly enhance its performance. Some future recommendations may include the following:

- Further research should focus on improving the interpretability of AI/ML-based soft sensor models;
- Given the dynamic nature of industrial processes, the potential for real-time updating or “online learning” in the context of soft sensors should be explored;
- The model’s ability to handle inconsistent and erroneous data, known as “dirty data”, should be further improved;
- The scalability of the soft sensor model should be further tested using data from a variety of real-life refinery operations;
- The use of advanced deep learning techniques should be further explored to develop more comprehensive, iterative, and adaptable solutions for predicting and optimizing component concentrations within distillation systems;
- The high applicability and potential for replication of the soft sensor model should be tested in similar industrial scenarios.

## Figures and Tables

**Table 1:** Main variable descriptive statistics.

Variable	Units of Measurement	Minimum Value	Maximum Value	Average Value	Standard Deviation
C2	% (mol/mol)	0.000	2.576	0.872	0.529
C5	% (mol/mol)	0.206	0.848	0.533	0.099
Energy consumption	kW	6992	12,240	9279	488
Dehydrator temperature	°C	67.078	73.242	69.965	0.904
Debutanizer pressure	(kg/cm^2^)	10.924	11.076	11.002	0.029
Reheater debutanizer flow	(m^3^/d)	2569	7025	4609	541
De-ethanizer temperature	°C	48.606	76.713	61.690	3.894
Desulfurizer pressure	(kg/cm^2^)	14.653	17.007	16.746	0.349
Reheater de-ethanizer flow	(m^3^/d)	1195	5810	2210	406

**Table 2:** C2, C5, and Q evaluation metrics for the ANN and RFR soft sensor models.

Model	Variable	Units of Measurement	RMSE	MAE	R^2^	Average Value	Standard Deviation
**ANN**	C2	% (mol/mol)	0.238	0.172	0.7976	0.872	0.529
	C5	% (mol/mol)	0.079	0.061	0.3618	0.533	0.099
	Q	kW	249.17	193.77	0.7396	9279	488
**RFR**	C2	% (mol/mol)	0.1108	0.062	0.9561	0.872	0.529
	C5	% (mol/mol)	0.0404	0.025	0.8346	0.533	0.099
	Q	kW	53.799	33.562	0.9878	9279	488

## Data Availability

Data sharing is not applicable to this article, due to privacy restrictions.
